# Seasonal Mortality of Wild Atlantic Menhaden (*Brevoortia tyrannus*) Is Caused by a Virulent Clone of *Vibrio (Listonella) anguillarum*; Implications for Biosecurity along the Atlantic Coastal United States

**DOI:** 10.1155/2024/8816604

**Published:** 2024-04-12

**Authors:** Jan Lovy, Luke R. Iwanowicz, Timothy J. Welch, Bassem Allam, Rodman G. Getchell, Sabrina Geraci-Yee, Chris Good, Jeremy Snyder, Clayton D. Raines, Nilanjana Das

**Affiliations:** ^1^U.S. Geological Survey, Western Fisheries Research Center, Seattle, WA 98115, USA; ^2^Office of Fish and Wildlife Health and Forensics, New Jersey Fish and Wildlife, Oxford, NJ 07863, USA; ^3^U.S. Geological Survey, Eastern Ecological Science Center, Kearneysville, WV 25430, USA; ^4^U.S. Department of Agriculture/Agricultural Research Service, National Center for Cool and Cold Water Aquaculture, Leetown, WV 25430, USA; ^5^School of Marine and Atmospheric Sciences, Stony Brook University, Stony Brook, NY 11794–5000, USA; ^6^Department of Microbiology and Immunology, College of Veterinary Medicine, Cornell University, Ithaca, NY 14853, USA; ^7^New Jersey Department of Agriculture, Animal Health Diagnostic Laboratory, Ewing, NJ 08628, USA

## Abstract

Atlantic menhaden are a highly migratory marine species in the Eastern United States that suffer from seasonal chronic mortality. Affected fish show neurologic signs referred to as spinning disease, including circling at the surface and erratic corkscrew swimming before death. We investigated three similar menhaden mortality events consistent with spinning disease in coastal New Jersey and New York between 2020 and 2021 to understand the cause. A unique strain of *Vibrio (Listonella) anguillarum* (serogroup O3) was detected regularly in high loads, particularly in the brains of moribund fish, by both metagenomics and bacterial isolation. The most common histopathological changes in moribund fish were hemorrhagic meningitis, encephalitis, pyknosis, and karyorrhexis of hematopoietic tissues in the kidney and spleen. Whole genome sequencing of isolates from moribund fish representing a wide spatial and temporal range showed that they were nearly identical clones, suggesting it to be a pathogenic strain circulating in the population. Though *V. anguillarum* is believed to be the main pathogen associated with spinning disease and mortality, *Yersinia ruckeri* (serotype O1) was isolated from smaller numbers of fish. Considering the highly migratory nature of Atlantic menhaden throughout the eastern United States and their use as bait for other fisheries, these findings identify potential biosecurity challenges that should be considered in Atlantic salmon aquaculture, fisheries, and emerging marine aquaculture in the region.

## 1. Introduction

Atlantic menhaden, *Brevoortia tyrannus*, are a schooling, migratory clupeid fish common in coastal waters spanning from Florida to Nova Scotia, Canada [1]. They are a critical forage species for game fish, including striped bass *Morone saxatilis* and bluefish *Pomatomus saltatrix*, as well as coastal birds and mammals in the region. Their importance as forage species in the ecosystem has led to stock management using ecological reference points to ensure the stability of the population while also considering interactions with predators and prey in the ecosystem [2, 3]. Atlantic menhaden, hereafter referred to as menhaden, also comprises a valuable commercial fishery, which is the largest by weight of any species in the U.S. Atlantic. Though historically comprised of a reduction fishery, since 2000, the use of menhaden as bait for commercial crab, lobster, and hook-and-line fisheries has increased [4]. The high biomass of menhaden throughout coastal areas along the eastern United States and their commercial use as bait highlights the importance of understanding disease in this species to better track disease-associated mortality and to identify biosecurity threats for salmonid aquaculture and for an emerging offshore aquaculture industry [5].

Mortality in menhaden is frequently reported in the mid-Atlantic Bight, particularly off the coasts of New Jersey and New York, USA. Acute episodes related to hypoxia from dense schools of fish, along with the presence of other stressors, are well documented [6]. Additionally, chronic-duration mortalities, clearly distinct from hypoxic episodes, have been reported as early as the 1950s [7, 8]. Affected fish have become known as “spinners” based on their exhibited neurologic signs, including circling at the water surface and erratic swimming [8]. Though early speculations believed these signs were associated with environmental factors [8], it was later suggested that they were in fact caused by an infectious disease. An infectious pancreatic necrosis (IPN)-like virus (aquabirnavirus) was isolated from affected fish, and experimental studies indicated that this virus caused “spinning disease,” suggesting this virus to be the primary etiology of these mortality events [9].

Since at least 2015, similar seasonal mortalities have become apparent off the coasts of New Jersey and New York, with 2020 and 2021 having particularly severe mortality events; thus, these were the focus of our investigation. The importance of understanding the cause of these mortalities is highlighted by the considerable public concern related to large numbers of dead menhaden in estuaries and rivers within highly populated areas. These have required large, coordinated clean-up efforts to dispose of the dead fish. Here we show that these recent epizootics were associated with a bacterial etiology, specifically the bacterium *V. anguillarum*, and that fish were also reservoirs of *Yersinia ruckeri*.

Both bacteria are known fish pathogens. Vibriosis, caused by *V. anguillarum*, is a problematic disease causing hemorrhagic septicemia best known in farmed marine finfish, though also reported in shellfish and crustaceans [10]. The bacterium was first isolated from European eels *Anguilla anguilla* suffering from “red pest” [11], a condition dating back to the 1700s. The bacterium was not detected in North America until its isolation in 1953 from diseased Pacific salmon [12]. It has since been recognized as a major disease of salmonids and other marine finfish. Although best known as causing disease in farmed fish, there have been documented mortalities attributed to *V. anguillarum* in wild fish, for example, in the sand-smelt *Atherina boyeri* in Greece [13]. *Y. ruckeri* is a bacterium responsible for enteric redmouth disease, highly pathogenic and first reported in rainbow trout *Oncorhynchus mykiss* [14], and now also known to cause disease in other non-salmonid fish species [15]. Herein, we characterize the bacteria of these genera from seasonal epizootics of menhaden to address health and biosecurity concerns in fisheries and aquaculture related to these pathogens in the Atlantic coastal region of the eastern United States.

## 2. Materials and Methods

### 2.1. Screening Menhaden for Viral Agents (2015–2020)

Since 2015, several chronic-duration menhaden mortalities have occurred in which fish exhibited neurologic signs, including erratic swimming and circling at the surface. The disease signs were consistent with “spinning disease,” believed to be caused by an IPN-like virus [9]. To screen for virus detection using cell cultures, brain, kidney, and spleen were aseptically collected from these mortality events and tested with cell lines and assays summarized in Table 1. Briefly, tissues were homogenized in nine volumes of Hank's Balanced Salt Solution (HBSS), followed by centrifugation. The supernatant was diluted 1 : 1 with antibiotic media and incubated for a minimum of two hours. Cells were grown in 24-well plates, and for each sample, 100 *µ*l of the centrifuged supernatant was added into duplicate wells onto freshly seeded cells (within 24 hr). Cells were monitored every 3 days for a total of 2 weeks, followed by the blind passage of samples onto freshly grown cells and further monitoring for 2 weeks. If no cytopathic effects occurred within the 4-week period, the samples were considered negative. If cytopathic effects (CPE) were observed, the homogenate from the well with CPE was passed onto fresh cells, and a sample was collected into a microcentrifuge tube and frozen at −80°C for molecular analysis. The passaged homogenate was monitored for CPE as described above. Kidney and brain tissue homogenates were also stored at −80°C for further molecular screening directly from the tissue.

Because of the neurologic signs exhibited, select fish were screened for the viral nervous necrosis virus (VNNV). During mortality in May 2020, seven menhaden showing “spinning” behavior were initially screened as described above using viral cell culture assays. The purified viral preparations of the brain were used to screen for VNNV with real-time PCR using a validated assay described by Panzarin et al. [16]. RNA was extracted from the homogenates using the MagMAX-96 Viral RNA Isolation Kit, automated on the Kingfisher Flex. cDNA was generated from extracted RNA using the High Capacity cDNA Reverse Transcription Kit, and real-time PCR for VNNV was run according to a previously reported protocol [16], except the Applied Biosystems 7500 Fast Real-Time PCR platform was used. All equipment and reagents listed above were sourced from Thermo Fisher Scientific, Waltham, MA, USA.

Samples were screened for IPN-like viruses or other viral etiologies. Cell culture of BF-2 cells exhibiting cytopathic effects from June 2015 to May 2020 were prepared for conventional PCR and NGS. RNA was extracted using a Total RNA I (Omega Bio-Tek, Norcross, GA, USA) kit per manufacturer protocols with on-column DNase digestion. RNA was synthesized into cDNA for conventional PCR using a High-Capacity cDNA Reverse Transcription Kit (Thermo Fisher, Waltham, MA, USA). Primers, VP1F—GTTGATMMASTACACCGGAG and VP1F—AGGTCHCKTATGAAGGAGTC, targeting a conserved region of segment B, were used [17]. The PCR reaction included 9 *µ*l of NF water, 13 *μ*l of GoTaqGreen (Promega, Madison, WI, USA), 10 *µ*m each of the forward and reverse primers, and 1 *µ*l of cDNA. The thermal cycling profile was 95°C for 5 min, 95°C for 30 s, 57°C for 30 s, 72°C for 40 s, for 30 cycles. Final extension at 72°C for 5 min, with final holding at 4°C. The products of the reaction were visualized on an agarose gel.

Given that previous descriptions of the IPN-like virus associated with spinning disease did not include genomic sequence data, we utilized *de novo* sequencing methods to not assume sequence identity. Total RNA from BF-2 cells inoculated for 96 hr with tissue lysate from clinically affected fish from 2015 to 2020 with suspected CPE and control cells was enriched for non-ribosomal RNA using a Ribo Zero kit as described above. RNA enriched for non-ribosomal RNA was then quantified using a Qubit, and a sequencing library was constructed using the NEBNext UltraTM RNA library kit for Illumina according to manufacturer protocols (NewEnglandBio Labs, Ipswich, MA, USA). The library was indexed to identify sequences from control and inoculated treatments, quantified using the KAPA Library Quantification Kit for Illumina (Roche, Indianapolis, IN, USA), and 10 pM of library was sequenced on a MiSeq (2 × 101 PE; Illumina).

We used the CLC Genomics Workbench (CGWB; v21.0.3, Qiagen) to trim and assemble reads using automatic kmer and bubble-size selection. All contigs were queried via blastx into a curated, in-house sequence database populated with the current viral protein records from the NCBI repository to identify putative viral contigs. In addition, we mapped unassembled short reads to the refseq IPNv segments A (NC_001915) and B (NC_001916) with a graded stringency of 80%–100% for targeted screening of short reads.

### 2.2. 2020 Fall Menhaden Mortality Investigation

#### 2.2.1. Fish Collection and Tissue Sampling

**Figure 1:**
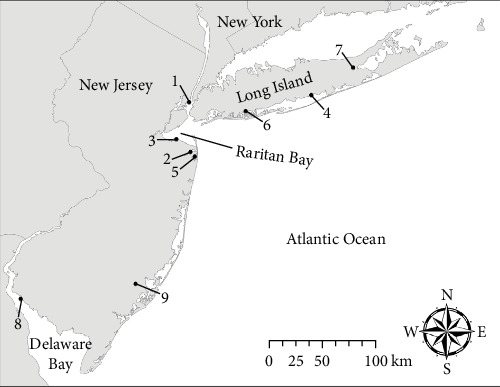
Map showing the collection locations of moribund Atlantic menhaden *B. tyrannus* from mortality events in 2020 and 2021. The location numbers correspond with further sampling and biological information in Tables 1 and 2.

Moribund menhaden (*n* = 30) were collected using a long-handled dip net from Raritan Bay sites around Liberty State Park, New Jersey (Figure 1) on 01 Dec 2020. Water temperature and dissolved oxygen (DO) were recorded at the time of sampling. Fish were euthanized, transferred on ice to the Pequest Aquatic Animal Health Laboratory, and stored overnight on ice. Fish necropsy and sample collection occurred approximately 15 hr following the collection of moribund fish. Fish measurements included total length (cm) and weight (g); Fulton's body condition index (k-factor) was calculated for each fish (weight/TL^3^ × 100). During necropsy, tissue samples were collected aseptically for viral cell culture assays, and kidney and brain samples were separately collected into 2 mL Eppendorf tubes and frozen at −80°C. For viral cell culture assays, five-fish pools of brain and five-fish pools of kidney and spleen were collected from all fish and maintained at 4°C until processing, as described above.

Histopathologic samples, including pyloric ceca, intestine, liver, heart, spleen, anterior and posterior kidney, and brain, were dissected and fixed into 10% neutral-buffered formalin (NBF). Following fixation for a minimum of 48 hr, histologic samples were routinely processed for histology, including dehydration through an ascending series of ethanols, clearing in Shandon xylene substitute, and infiltration with paraffin wax. Tissues embedded in paraffin wax were sectioned at 5 *µ*m, mounted on glass slides, and stained with hematoxylin and eosin. Slides were examined and photographed with a Zeiss Axioplan-2 research microscope with a mounted Jenoptik Gryphax Arktur digital camera (Jena, Germany).

#### 2.2.2. Metagenomic Screening and Bacterial Isolation

Based on histopathologic evidence of bacterial infection in the fish, previously frozen pelleted tissue that resulted from virology processing was used as the source of DNA to screen for bacteria using metagenomics. Eight samples were processed, including four five-fish pools containing kidney/spleen and four five-fish pools containing brain. DNA was extracted using Indimag, a magnetic bead-based method, according to the manufacturer's directions. DNA concentration was measured using Qubit and normalized to 20 ng/*µ*l. 16S-EZ rDNA next generation sequencing library preparations and Illumina sequencing were conducted at Genewiz, Inc. (South Plainfield, NJ, USA). A sequencing library was prepared using a MetaVx 16S rDNA Library Preparation Kit (Genewiz, Inc., South Plainfield, NJ, USA). The DNA was used to generate amplicons covering the V3 and V4 hypervariable regions of bacterial and archaeal 16S rDNA. Indexed adapters were added to the ends of the 16S rDNA amplicons by limited-cycle PCR. DNA libraries were validated and quantified before loading. The pooled DNA libraries were loaded on an Illumina MiSeq instrument according to the manufacturer's directions (Illumina, San Diego, CA, USA). The samples were sequenced using a 2x 250 paired-end configuration. With the intent to identify the predominant bacterial taxa within the samples, the taxa with the highest sequence reads were identified and reported from the samples.

Bacterial cultures were taken from select frozen kidney samples from individual fish onto tryptic soy agar (TSA; MP Biomedicals LLC, Solon, USA) supplemented with 2% NaCl and incubated at 25°C for up to 72 hr. Bacterial colonies growing on TSA were identified using matrix-assisted laser desorption ionization-time of flight mass spectrometry (MALDI-TOF) at the New Jersey Department of Agriculture's Animal Health Diagnostic Laboratory. Following identification, single colonies from plate cultures were inoculated into tubes containing TSA broth supplemented with 2% NaCl and incubated at 25°C. Following 24–48 hr of growth, the broth cultures were mixed with an equal volume of 80% sterile glycerol, transferred to 2 ml microcentrifuge tubes, and frozen at −80°C.

### 2.3. 2021 Spring and Winter Menhaden Mortality Investigations

#### 2.3.1. Fish Collection and Sampling

Two separate mortalities were investigated in 2021, a large mortality estimated to impact hundreds of thousands of fish in the spring and a separate smaller mortality in the winter that impacted hundreds of fish and was localized to a small tributary of the Mullica River, NJ, USA.

In the spring mortality, moribund fish were collected from New Jersey locations during three time periods, representing an early sample (30 March), midpoint (22 April to 26 April), and when mortality was subsiding (12 May to 17 May) (Table 2). When possible, water temperature and DO were recorded during collection. Moribund fish were captured with a dipnet, euthanized with an overdose of sodium bicarbonate buffered MS-222 (200 mg L^−1^) and transported on ice to the Pequest Aquatic Animal Health Laboratory. Necropsy and sample collection occurred immediately upon return from the field (2–4 hr after collection), except for fish collected on 26 April which were held overnight on ice and sampled the following morning. Fish total length (cm) and weight (g) were recorded, and the body condition factor (k) was calculated. Organ samples were collected for histology from all fish, as described above, except for the 20 fish collected on April 26. From those 20 fish, samples for the viral cell culture assay were collected and processed as described above. Bacteriological samples were taken from all fish, as further described in detail below.

From this same mortality event, fish were collected from Long Island by the New York Department of Environmental Conservation. Collection locations and biological data are summarized in Table 2 and Figure 1. Fish were transported on ice to the Marine Animal Disease Laboratory at Stony Brook University for bacteriological processing on the same day.

In the winter mortality in Port Republic, New Jersey, a total of five moribund fish were collected, two of which were collected and frozen on December 22 and three collected on December 28, held on ice, and necropsied several hours after collection. Histology was conducted as described above on the three freshly processed fish. Bacteriology by plating the kidney and brain to determine colony-forming units (CFU)/g of tissue was done on all five fish, as further described below.

#### 2.3.2. Bacteriology

Bacteriology conducted at the Pequest Aquatic Animal Health Laboratory included aseptically streaking the brain and kidney of each fish onto freshly made TSA plates supplemented with 2% NaCl. Plates were incubated for up to 72 hr at 25°C. After 48 hr, plates were examined, and a relative bacterial growth intensity was recorded with the following criteria: Light growth was few, clearly isolated colonies (<20) along the streak line; moderate growth was over 20, but under 100 colonies clearly isolated from each other along the streak line; and heavy growth had coalesced colonies uniformly covering the entire streak line. For a more accurate estimation of infection intensity, CFU/g of tissue was determined in fish that were collected during the late sampling period of the spring mortality (*n* = 12) and the fish collected from the winter mortality (*n* = 5). Briefly, small pieces of kidney and brain (<100 mg) were aseptically dissected and transferred to separate 2 ml screw-top microtubes. The tissues were diluted with sterile phosphate buffered saline (PBS) (10 *µ*l/mg tissue) and homogenized using a Mini Beadbeater (Biospec Products, Bartlesville, OK, USA) with 5 mm glass beads. Serial dilutions were made from the homogenate. One hundred microlitres of the original homogenate and the dilutions were plated on fresh TSA with 2% NaCl, spread with a sterile plate spreader, and incubated for up to 48 hr at 25°C. Colony counts were done using a Zeiss Stemi stereomicroscope (Carl Zeiss, Germany). Colony counts were calculated as CFU/g of tissue. Single bacterial colonies were subcultured onto either TSA with 2% NaCl or blood agar. These pure cultures were identified by MALDI-TOF, subcultured into TSA with 2% NaCl broth, and cryopreserved as described above.

From the spring mortality, a total of 14 fish from New York were submitted to the Marine Animal Disease Laboratory, Stony Brook University. Fish were measured, cleaned, and aseptically dissected. Brain and kidney tissues were individually swabbed with a sterile loop and plated onto brain heart infusion (BHI) agar with 2% salt and thiosulfate-citrate-bile salts-sucrose (TCBS) agar (BD, Difco). Tissue biopsies of the remaining brain and kidney were collected and stored at −80°C. Plates were incubated in the dark at room temperature and monitored for growth daily. Colonies were then cross-plated onto either TCBS or marine agar (BD, Difco) and subcultured until single colony isolates were obtained. Bacterial identification was determined by MALDI-TOF, as described above. Bacterial isolates were cultured in marine broth (BD, Difco) for 24–48 hr for DNA extraction and cryopreservation. Multiple replicates were cryopreserved in 20% glycerol (final concentration) using Mr. Frosty freezing containers (Thermo Fisher Scientific, Waltham, MA) and stored at −80°C.

To roughly determine temperature and salinity effects on plate growth, a single *V. anguillarum* isolate from the winter 2021 mortality was grown at various temperatures and salinities. Following re-isolation on TSA with 2% NaCl, a single colony was inoculated in Tryptic Soy Broth (Remel, Lenexa, KS, USA) containing 2% NaCl and incubated for 24 hr at 25°C. Four replicate plates were inoculated for each temperature (4, 15, 25, and 37°C); the quadrant streak plate method was used to inoculate and estimate bacterial growth on plates. For salinity treatments, TSA was prepared with 0%, 2%, 4%, or 6% added NaCl. Four replicates for each salinity were inoculated using the same method, and all were incubated at 25°C. Bacterial growth was enumerated with a semi-quantitative score every 24 hr up to 72 hr as follows: growth in quadrant 1 = 1+, quadrant 2 = 2+, quadrant 3 = 3+, and all four quadrants = 4+ growth.

### 2.4. Lipopolysaccharide Serotyping

#### 2.4.1. *V. anguillarum* Strains

For the production of serotyping antisera, bacteria were grown overnight in tryptic soy broth supplemented with 1% NaCl (TSBS) media at 24°C shaking at 200 rpm, harvested by centrifugation, and resuspended in sterile PBS, to which formaldehyde was added to a final concentration of 0.2%. After incubation overnight at 4°C, cells were again washed with PBS, adjusted to a concentration of 1.0 × 10^9^ cells/mL, and tested for sterility by plating onto TSBS. Polyclonal antisera were produced in New Zealand white rabbits by injection with the formalin-killed cells using the following injection schedule. Priming was accomplished by injection with 0.5 mL of cells mixed 1 : 1 with complete Freund's adjuvant. This was followed by injection with 0.5 ml of cells mixed 1 : 1 with incomplete Freund's adjuvant 25, 46, and 74 days later. Serum was then collected 105 days after the first immunization. All procedures were performed by Pacific Immunology (Ramona, CA, USA). Lipopolysaccharide (LPS) was prepared using the method of Hitchcock and Brown [18] and resolved by SDS-PAGE with a 15% Criterion Tris-Tricine precast gel as directed by the manufacturer (Bio-Rad). LPS was then electroblotted onto nitrocellulose membranes, blocked for 1 hr at room temperature, washed in PBS, and then incubated for 2 hr at room temperature with a 1/5,000 dilution of the antiserum preparations described above. LPS antibodies were then detected using the Immun-Blot Opti-4CN detection kit as directed by the manufacturer (Bio-Rad). All incubations and washes were performed using the Bandmate Automated Western Blot Processor (Invitrogen).

#### 2.4.2. *Y. ruckeri* Strains

The serotype was assessed in *Y. ruckeri* by slide agglutination using antiserum raised against strain CSF087-82 prototype 01 strain [19]. The serotyping antiserum for *Y. ruckeri* was produced as described above for the *V. anguillarum* antiserum. Agglutinations were performed on glass slides with antisera diluted 1 : 10 with PBS. The presence or lack of agglutination was determined 60–90 s after a small loopful of bacteria was mixed with 50 *μ*l of diluted antiserum. Autoagglutination was discounted by mixing a loopful of bacteria with 50 *μ*l of PBS. A culture of CSF087-82 was used as a positive control and pre-immune serum as a negative control. The serotype was also determined by sensitivity to a serotype O1-specific bacteriophage using a double-agar plague assay [20].

### 2.5. Whole Genome Sequencing and Multilocus Sequence Typing of V. anguillarum

A subset of isolates confirmed as *V. anguillarum*, representing a wide spatial and temporal scale, were selected for genome sequencing. A total of 11 isolates were grown overnight in TSBS at 24°C, shaking at 200 rpm, harvested by centrifugation, washed in sterile PBS, and the equivalent of 10 OD_600_ were suspended in DNA/RNA shield. Suspended cells were shipped to Plasmidsaurus (Eugene, OR, USA) for extraction and Oxford Nanopore long-read sequencing. DNA was extracted using the MasterPure DNA Purification Kit (Lucigen, WI, USA) and prepared for Illumina sequencing for genome polishing. In short, extracted bacterial DNA was quantified using a Qubit 4.0 fluorometer (Invitrogen, CA, USA) and then normalized to a concentration of 0.2 ng/*µ*l. Five microlitres of the normalized product were used as starting material for NGS library prep. An Illumina Nextera XT Library Preparation Kit (Illumina, CA, USA) was used according to the manufacturer's reference guide (Doc # 15031942 ver. 5) for MiSeq preparation. Normalization of the final library was performed with the Illumina bead-based normalization method and pooled as described in the BBN Loading Concentrations Exceptions Table 2 in the MiSeq System Denature and Dilute Libraries Guide (Doc # 15039740 ver. 10). Pooled libraries were then run for 2 × 301 cycles and loaded with a 15% PhiX (12.5 pM) spike.

Assembly of long reads was performed by removing the lowest-quality 5% fastq reads using default parameters in Filtlong v0.2.1 [21]. Reads were downsampled to 250 Mb using Filtlong to create a draft scaffold of the assembly using Miniasm v0.3. Reads were then downsampled to ∼100x coverage, and a Flye v2.9.1 assembly was conducted with parameters selected for high-quality ONT reads. This assembly was polished using Medaka v1.7.2. The assembly was further polished with Illumia MiSeq reads. Paired-end reads were imported into CGWB, trimmed using default settings, and then mapped to draft chromosomes. Draft genomes were reordinated such that the start codon of the dnaA gene was at base 100 and annotated using the DFAST workflow [22]. The whole genome multiple alignment of chromosome 1 was performed using the CGWB whole genome analysis tool. Average nucleotide identity (ANI) comparisons were conducted based on coding sequence (CDS) using default settings for draft chromosome 1 sequences of menhaden isolates, strain NB10 (serotype O1), strain M3 (serotype O1), strain J360 (serotype O2), strain VIB12 (serotype O2), strain PF4 (serotype O3), strain PF7, strain PF430-3 (serotype O3), and strain CNEVA NB11008 (serotype O3) to estimate relative genomic diversity. Heat maps were constructed using Manhattan distances and completed linkage of predicted CDS. We then performed multilocus sequence typing using autoMLST [23]. The concatenated alignment of autoMLST-selected loci was imported into Geneious Prime 2022.2.1 (Biomatters, Inc., Boston, MA, USA) for further sequence analysis. Specific non-*Vibrio anguillarum* sequences were culled from the original output of 50 to refine the resolution of the phylogenetic analysis. Sequences were realigned using MUSCLE, and phylogenetic analysis was conducted using Mr. Bayes (General Time Reversible model; total chain length = 2,00,000; subsample frequency = 200; burn-in = 20,000) [24]. *Vibrio ordalii* ATCC 33509 was set as an outgroup. The sequence demarcation tool v1.2 was utilized to visualize pairwise sequence identity across concatenated sequences of the 43 vibrio strains, including the 11 isolates from the current study [25].

### 2.6. Experimental Infection of Atlantic Salmon with V. anguillarum

Atlantic salmon were obtained as diploid, all-female-eyed eggs from a certified disease-free supplier (Stofnfiskur, Iceland). Stock and test fish were maintained at 14°C in fresh water under flow-through conditions and fed a standard diet 7 days per week. The National Center for Cool and Cold Water Aquaculture Institutional Animal Care and Use Committee reviewed and approved all experimental procedures used in this study. At the time of the challenge, fish were 12–15 g and 20 fish were used per dose tested. Fish were inoculated with test strains by intraperitoneal (IP) injection in a volume of 0.1 ml under sedation with buffered MS-222 (90 mg L^−1^) using a 0.5 ml Tuberculin syringe (27G 1/2-inch needle). Postinfection fish were monitored for 28 days, and mortalities were removed and recorded daily. Mortality due to *V. anguilarum* was confirmed by microbiological analysis of kidney tissue in at least 20% of the mortalities/day. For the challenge, bacterial strains were grown overnight in TSBS at 24°C with shaking at 200 rpm, washed with PBS, and the optical density (600 nm) adjusted to the approximate doses. The actual CFU used was confirmed by direct plate counting. Kaplan–Meier survival plots were created using the GraphPad Prism 9 software package (GraphPad Software Inc., San Diego, CA, USA).

## 3. Results

### 3.1. Viral Screening from 2015–2020

Viral cell culture assays were negative for viral agents; the results are summarized in Table 1. Kidney, spleen, and heart homogenates from fish screened from June 2015 (*n* = 3) and brain homogenates from fish screened from May 2020 (*n* = 7) showed cytopathic effects (CPE) in BF-2 and CHSE-214 cells. Cells appeared hypertrophic, rounded up, and detached from the monolayer, leading to holes in the monolayer and stacking of cells on the periphery. When these cell homogenates were passed to newly seeded cells, no CPE occurred in the following passage. Bioinformatic analysis of Illumina short reads did not provide evidence of the IPN virus, nor did other presumptive viral candidates identified as associated with CPE. A circovirus-like virus was found with a very low copy number in the BF-2 cells. Primers were designed to amplify this virus in subsequent tissue samples, but all other samples were negative for this target, and thus it was considered an incidental finding and not associated with disease in menhaden (data not shown). All samples were also negative for VNNV. Considering that CPE was absent upon subsequent passage and the virus was not detected using sensitive molecular methods, it is unlikely that it was related to a viral etiology and more likely caused by the presence of bacterial cytotoxins. This was consistent with seeing higher CPE in the brain samples, which contained the highest amount of bacteria.

### 3.2. 2020 Fall Menhaden Mortality Investigation

#### 3.2.1. Mortality Description, Gross, and Histopathologic Observations

Chronic mortality of menhaden was first reported in November and occurred through mid-December 2020, with fish exhibiting erratic swimming behavior and often swimming in tight circles near the water surface, typical signs previously described for spinning disease. Other fish were lethargic and near the water surface. Schools of fish with normal behavior were observed in the same region. Total mortality could not be accurately estimated, though it likely impacted tens of thousands of fish. Fish collection sites (Figure 1) and biological data (Table 2) are summarized.

**Figure 2:**
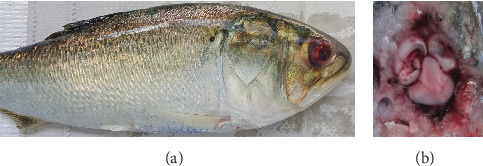
Gross findings from moribund *B. tyrannus* with *V. anguillarum* collected in December 2020. (a) Fish with unilateral eye hemorrhage and no other external lesions. (b) Hemorrhage and congestion of the brain; notice the bloody fluid within the brain cavity.

External lesions were mostly absent or mild in the affected fish.  Table 3 contains a summary of gross and histopathologic findings. Infection with the anchor worm, *Lernaeenicus radiatus*, a copepod parasite that penetrates the skin and anchors into the muscle of the fish, ranging from 0 to 7 parasites/fish, was noted. Infection with the parasite was associated with ulceration and muscular degeneration at the infection site. The most common external finding in the fish was eye hemorrhage (Figure 2(a)). Internally, the organs and viscera appeared dark due to congestion and hyperemia. The gastrointestinal tract was empty and contained fluid, and the gall bladder was enlarged, indicating that fish were not actively feeding. This is consistent with the slightly depressed *k*-factors. The most prominent finding in gross necropsy was congestion and hemorrhage in and around the brain (Figure 2(b)).

**Figure 3:**
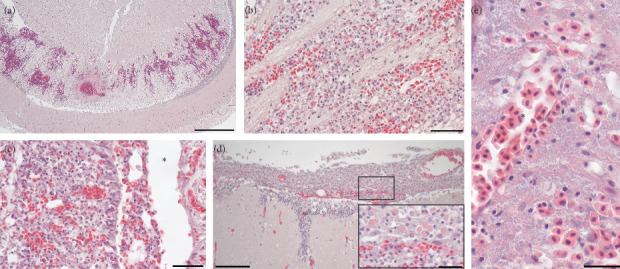
Brain histopathology of moribund *B. tyrannus* with *V. anguillarum*. (a) Optic tectum with congestion and hemorrhage within the *stratum periventriculare* layer and dilated blood vessels with perivascular fibrin deposition. Bar = 500 *µ*m. (b) Hemorrhagic encephalitis containing mainly eosinophilic granule cells. Bar = 50 *µ*m. (c) Hindbrain with cell degeneration and hemorrhage. Red blood cells and degenerated cells have sloughed into the fourth ventricle (*⁣*^*∗*^). Bar = 50 *µ*m. (d) Hemorrhagic meningitis; inset shows large numbers of eosinophilic granule cells. Bar = 100 *µ*m; inset bar = 25 *µ*m. (e) Hindbrain with hemorrhage and massive amounts of bacteria surrounding a blood vessel (*⁣*^*∗*^). Bar = 20 *µ*m.

Histopathologic lesions are summarized in Table 3. The most common were hemorrhagic lesions in the brain (Figure 3). Lesions occurred in the optic tectum and the hindbrain, and in some instances, heavy sloughing of degenerated and inflammatory cells occurred into the 4^th^ ventricle of the hindbrain. In the optic tectum, hemorrhage occurred within the periventricular layer adjacent to the 3^rd^ ventricle. In this region, the blood vessels were dilated, hemorrhage occurred, and fibrous material was deposited around the blood vessels (Figure 3(a)). Degenerated cells and encephalitis comprised of eosinophilic granule cells (EGCs) were present in the brain tissue (Figure 3(b)). The hindbrain was hemorrhaged and contained degenerated cells and an inflammatory infiltrate, which sloughed into the 4^th^ ventricle (Figure 3(c)). Meningitis occurred with an infiltrate that contained EGCs (Figure 3(d)). In the hindbrain of two fish, large numbers of bacteria occurred throughout the tissue (Figure 3(e)), with some macrophages engorged with bacteria.

**Figure 4:**
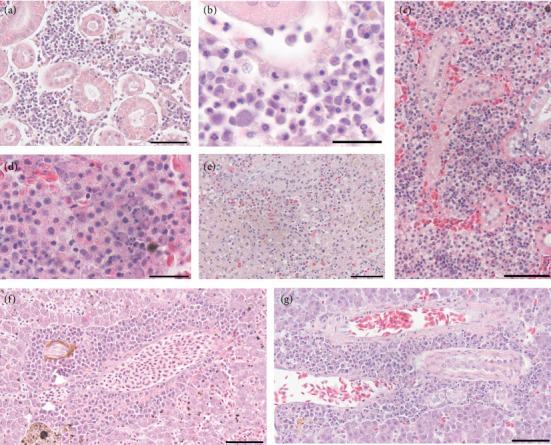
Histopathology in the internal organs of moribund *B. tyrannus* with *V. anguillarum*. (a) Severe degeneration of hematopoietic cells in the kidney. Bar = 50 *µ*m. (b) Higher magnification of pyknotic and degenerated hematopoietic cells in the kidney. Bar = 20 *µ*m. (c) Kidney with severe infiltration of eosinophilic granule cells and degenerated hematopoietic cells. Bar = 50 *µ*m. (d) Higher magnification of eosinophilic granule cells, some of which appear degranulated, and hematopoietic cells that are enlarged and have pyknotic nuclei. Bar = 20 *µ*m. (e) Spleen with severe degeneration of leukocytes. Bar = 50 *µ*m. (f) Liver with an inflammatory infiltrate, predominantly eosinophilic granule cells, surrounding a blood vessel. Bar = 50 *µ*m. (g) Liver perivascular inflammatory infiltrate with degenerative changes, including swelling and pyknosis. Bar = 50 *µ*m.

Other tissues with lesions included the kidney, spleen, and liver (Figure 4), summarized in Table 3. In the kidney, diffuse hematopoietic cell degeneration occurred. The hematopoietic cells had pyknosis and karyorrhexis (Figure 4(a)–4(d)). Among the kidney hematopoietic cells were large numbers of EGCs, making up much of the cell composition (Figures 4(c) and 4(d)). The EGC granules were often free in the tissue, resulting from either the breakdown of the cells or degranulation. Splenic hematopoietic cell degeneration was present (Figure 4(e)). In the liver, the hepatic parenchyma was normal in all fish. Hepatic perivasculitis comprised of EGCs and macrophages was present (Figure 4(f)). Degenerative changes often occur in the inflammatory cells surrounding the blood vessels (Figure 4(g)).

#### 3.2.2. Metagenomic Analysis for Bacterial DNA

With histological evidence for bacterial infection, metagenomic analysis was done to determine the presence of bacterial species. Metagenomic analysis of the brain and kidney showed that sequences consistent with *V. anguillarum* occurred in all four pools of brain tissue, with high sequence reads between 33,437 and 40,198 (Table 4). Based on sequence reads, the expression of *V. anguillarum* was 35–43 times higher than other bacterial taxa identified in the brain, and the brain had heavy *V. anguillarum* expression compared to low expression in the kidney. Other bacterial taxa detected, particularly in the kidney, were in considerably lower concentrations than *V. anguillarum* in the brain (Table 4). For additional confirmation of *V. anguillarum* from the brain, bacteria were isolated from corresponding previously frozen brain samples onto TSA with 2% NaCl. The isolated bacteria were identified by MALDI-TOF as *V. anguillarum*.

### 3.3. 2021 Spring Menhaden Mortality

#### 3.3.1. Gross Necropsy and Bacteriological Results

**Figure 5:**
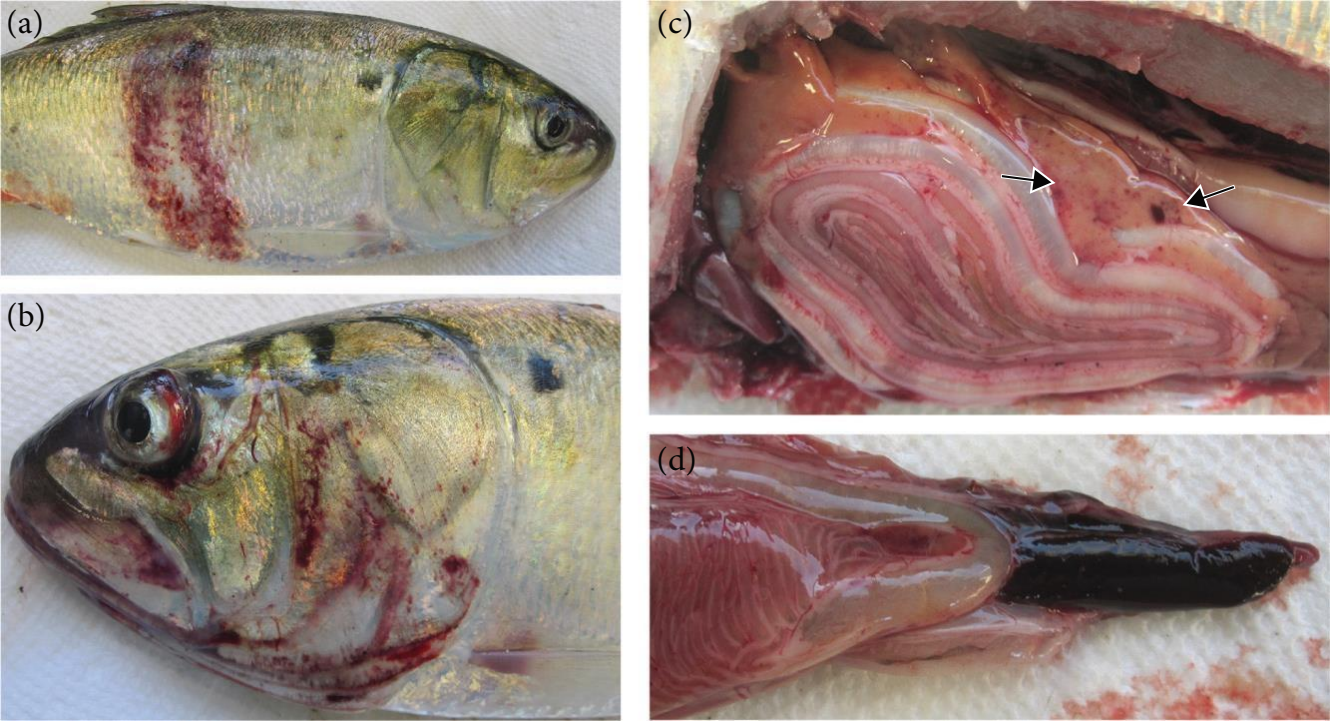
Systemic disease signs of moribund *B. tyrannus* collected during the early stages of mortality in March 2021. (a) Fish with large hemorrhagic skin lesions leading to ulceration in some areas. (b) Hemorrhagic exophthalmia and dermal hemorrhage on the head, mouth, and operculum. (c) Multifocal hemorrhages in the liver (arrows). (d) splenomegaly.

A general summary of the gross, bacteriological, and histopathologic findings is summarized in Table 3. Over the mortality period, *V. anguillarum* was detected in 49/61 fish (Table 3). In the early sampling period, external disease signs included severe hemorrhage on the head, opercula, and body surface (Figure 5). *L. radiatus* (anchor worm) ranged from 0 to 12 parasites per fish. Internally, fish with external hemorrhage had splenomegaly, multifocal hemorrhages in the liver (Figure 5), pancreas, and fluid-filled intestines. Based on bacterial culture, seven of these fish had heavy growth of pure *V. anguillarum* from both kidney and brain, and one fish had moderate growth in the brain and no growth in the kidney. Two fish were co-infected with *Pseudomonas aeruginosa* and *Vibrio tasmaniensis*. Two fish were negative for *V. anguillarum*, with one fish positive for *Aeromonas* sp. (definitive species ID not available) in the brain and kidney.

In the mid-phase of mortality, *V. anguillarum* was isolated from 26 out of 32 moribund fish, with 24 of these being pure isolations of the bacterium. Gross and microscopic lesions are described in Table 3. *V. anguillarum* isolation was more frequent and had higher growth from the brain when compared to the kidney in nearly every instance. Ten fish had heavy bacterial growth from the brain and no growth in the kidney; seven fish had heavy growth from the brain and light growth from the kidney; two fish had heavy growth from the brain and moderate growth from the kidney; two fish had moderate growth from the brain and no growth from the kidney; two fish had light growth in the brain and no growth in the kidney; and one fish had light growth from both the brain and kidney. Two fish were co-infected with *Y. ruckeri*. In one of these fish, *V. anguillarum* was purely isolated from the brain with heavy growth, whereas pure isolation of *Y. ruckeri* occurred from the kidney with moderate growth. In the second fish, mixed colonies of *V. anguillarum* and *Y. ruckeri* occurred in the brain, and pure isolation of *Y. ruckeri* occurred in the kidney with moderate growth. Lastly, a single fish had pure *Y. ruckeri* isolated from both the brain and kidney.

In the late phase of the mortality, fewer moribund fish were present than at the other sampling times, indicating the mortality was subsiding. External lesions were rare (Table 3), and *L. radiatus* was attached in the musculature, ranging from 0 to 23 parasites per fish. Of 12 fish, *V. anguillarum* was detected in eight fish, four with pure isolations and four with mixed infections. Other bacteria isolated included *Vibrio scophthalmi, Serratia plymuthica*, and *Aeromonas salmonicida*. *V. anguillarum* was the predominant bacterium isolated from both the brain and kidney, with mean CFU counts in the brain of 1.64 × 10^7^ CFU/g (range, 0 – 8.19 × 10^7^ CFU/g) and in the kidney with a mean of 5.32 × 10^5^ CFU/g (range, 0 – 3.38 × 10^6^ CFU/g). Three moribund fish yielded pure isolations of *Y. ruckeri* with high colony counts from the brain and kidney. Mean colony counts for *Y. ruckeri* from the three fish were 2.79 × 10^6^ (range 1.19 × 10^5^ – 7.04 × 10^6^ CFU/g) in the brain and 2.77 × 10^5^ (range, 2.3× 10^3^ – 4.72 × 10^5^ CFU/g) in the kidney.

A total of 14 fish were processed at the Marine Animal Disease Laboratory at Stony Brook University, representing three different locations and time points in the Long Island, NY, USA region (Figure 1). Of eight fish collected on April 13, 2021, three had pure *V. anguillarum* isolated from the brain and were negative in the kidney; one fish had pure isolations of *V. anguillarum* from both the brain and kidney; *Y. ruckeri* was isolated from the brain and kidney of three fish; and a single fish was negative for bacterial growth. All three fish that were collected on May 10, 2021, yielded pure isolations of *V. anguillarum*, with two positive in the brain and kidney and one fish with isolation only from the brain. Finally, of the three fish collected on May 12, 2021, two yielded pure cultures of *V. anguillarum* from the brain only, and one fish had *Curtobacterium flaccumfaciens* isolated from the brain. Other findings during necropsy included the copepod parasite *L. radiatus* embedded in the muscle of 8/14 fish, ranging from 3 to 7 parasites per fish.

#### 3.3.2. Histopathological Findings

Histopathological findings were consistent with those observed in 2020; a summary of findings is in Table 3. Of note, the incidence of brain hemorrhage and meningitis was more frequent in the middle part of the mortality when compared to the early samples (Table 3). Hemorrhage was present in the periventricular layer of the optic tectum, where fibrin accumulated adjacent to blood vessels. Cell degeneration and sloughing into the third and fourth ventricles occurred (Figure 3). Other notable lesions in the early part of the mortality included abundant melanomacrophage centers throughout the spleen (seven fish); multifocal hemorrhages throughout the tissue (two fish); and severe diffuse hepatocellular necrosis (one fish). No significant lesions occurred in the heart, except for one fish with microsporidial sporophorocysts throughout the spongy layer of the ventricle. The gastrointestinal tract epithelium was frequently sloughed, with many necrotic cells within the lumen, which may be at least partially related to postmortem autolysis. Histopathology associated with the middle and late phases of mortality is summarized in Table 3. Of the two fish that yielded pure cultures of *Y. ruckeri*, one had only a mild perivascular infiltrate comprised of macrophages in the liver. The second fish had moderate numbers of granulomas throughout the spleen and kidney, along with multifocal hemorrhages and granulomas within the liver.

### 3.4. 2021 Winter Menhaden Mortality

Based on observer reports, mortality occurred in Mill Pond, a tributary of the Mullica River, NJ, USA (location 9 in Figure 1), between the middle and end of December 2021. Dying fish exhibited spinning behavior, like the previous menhaden kills. Site collection and biological data are summarized in Figure 1 and Table 2, respectively. During the time of the investigation on December 28, about 150 dead fish were observed along the banks. A summary of the gross, bacteriological, and histopathologic findings is included in Table 3. Pure bacterial cultures of a single colony type, all confirmed to be *V. anguillarum*, were isolated from all five fish. Considerably heavier bacterial loads occurred in the brain than in the kidney. All five fish had *V. anguillarum* in the brain, with a mean of 1.56 × 10^7^ CFU/g (range, 1.4 × 10^5^ – 5.6 × 10^7^ CFU/g). Fewer bacteria were isolated from the kidney with two fish with no bacterial growth, two fish with 200–300 CFU/g, and a single fish with 5 × 10^6^ CFU/g.

#### 3.4.1. Temperature and Salinity Effects on *V. anguillarum* Plate Growth

The *V. anguillarum* isolate grew successfully at a wide range of temperatures and salinities. For temperature, observations at 24 hr showed the heaviest growth at 25°C, followed by moderate growth at 37°C. At 15°C, limited growth was observed initially, but samples reached moderate growth levels comparable to 37°C after 48 hr. Although no bacterial growth was seen at 4°C after 72 hr, samples were allowed to incubate further, and low growth (quadrant 1+) was recorded after 10 days. For NaCl, this isolate grew successfully at all four levels but had the heaviest growth (quadrant 2+) with TSA without added NaCl and 2% NaCl, followed by moderate growth (quadrant 2+) at 4% and 6% after 72 hr.

### 3.5. Lipopolysaccharide Serotyping

**Figure 6:**
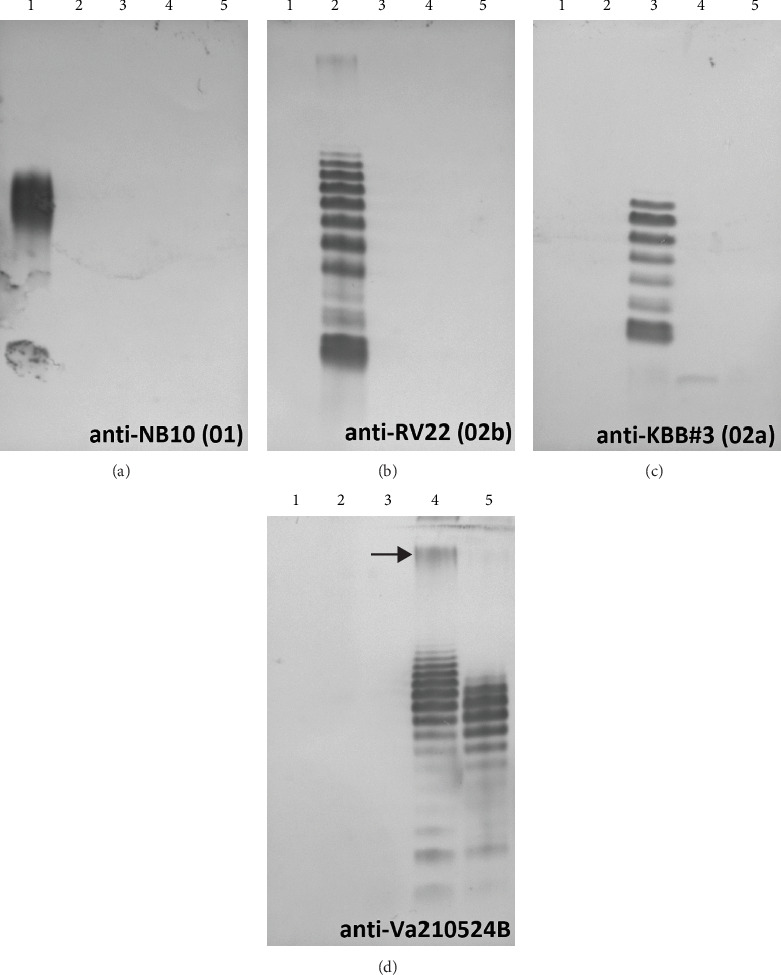
SDS-PAGE analysis of proteinase K-digested whole-cell lysates visualized by Western blotting using antiserum raised against serotype 01 (panel (a)), serotype 02b (panel (b)), serotype 02a (panel (c)), or menhaden strain Va210524B (panel (d)). Lanes: 1, NB10 (serotype 01); 2, RV22 (serotype 02b); 3, KBB#3 (serotype 02a); 4, Va210524B (menhaden strain); 5, ATCC 3,631,561 (serotype O3). Note that the menhaden isolate produced a proteinase K-resistant antigen (arrow), which is absent in the serotype O3-type strain. LPS was normalized to culture optical density.

Serotyping of *V. anguillarum* strains was performed by Western blotting of lipopolysaccharide extracts and probing with antisera raised against whole killed *V. anguillarum* cells of differing serotypes. LPS serotyping from a menhaden strain did not cross-react with serum specific to serotype O1, O2b, or O2a strains (Figure 6). However, antiserum raised against menhaden strain 21-5-24b cross-reacted with serotype O3 type strain (ATCC363156) and did not cross-react with serum raised against any of the other serotypes tested. Furthermore, LPS banding patterns were identical between the serotype O3 type strain and 21-5-24b, with the exception that 21-5-24b produced slightly higher molecular weight LPS and appeared to produce an additional proteinase K-resistant antigen absent in the serotype O3 type strain (denoted with an arrow in Figure 6). Thirty-five additional strains collected from the menhaden outbreaks were examined for cross-reactivity with serum raised against 21-5-24b, and the majority (31/36) displayed the O3 antigen LPS pattern (Supplemental materials Figure 1). These results demonstrate that the majority of *V. anguillarum* strains isolated from the menhaden outbreaks are serotype O3. Five strains did not cross-react with the O3 antiserum, and the serotype was not further determined. All *Y. ruckeri* isolates were highly motile, thus belong to biotype I. All 12 strains tested were uniformly serotype O1.

### 3.6. Molecular Typing of V. anguillarum

**Figure 7:**
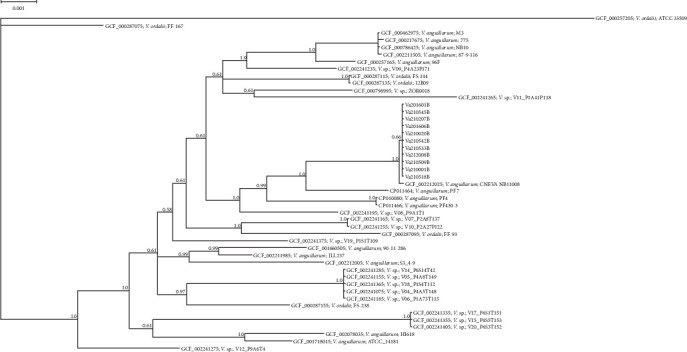
Phylogenetic relationships of *V. anguillarum* isolates to closely related strains based on multilocus sequence typing (MLST) of the 20 gene (18,408 bp). Branches are labeled with posterior probability. *V. ordalii* (ATCC 33509) was set as an outgroup.

**Figure 8:**
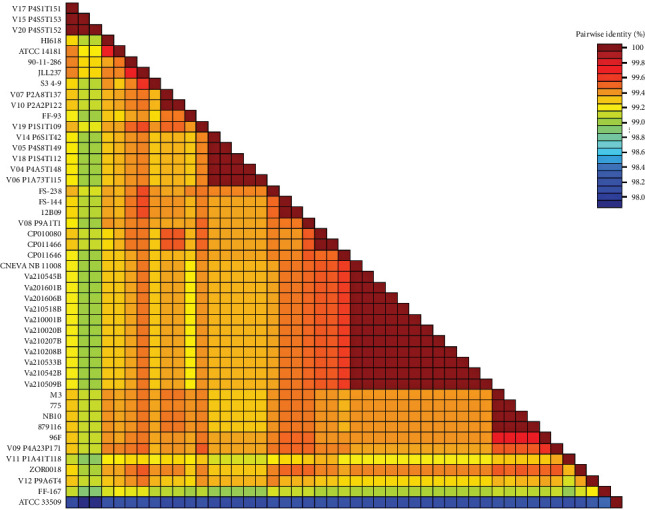
Pairwise identity matrix of concatenated gene sequences (20) used for multilocus sequence typing (MLST) of *V. anguillarum* isolates and closely related strains of *Vibrio spp*.

**Figure 9:**
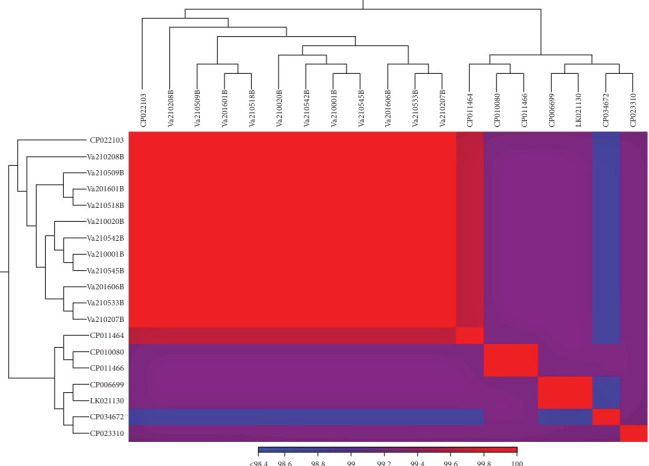
Average nucleotide identity (ANI) was determined for chromosome 1 across the bacterial isolates and representative serotypes 01, 02, and 03 genomes. A heat map was constructed using Manhattan distances and completed linkages of the predicted coding sequence.

Draft genome sequences of 11 *V. anguillarium* strains were assembled. The genomes of the isolates included two chromosomes consistent with those of representative *V. anguillarum* strains. No plasmids were identified. All sequence data were submitted to the NCBI under Bioproject: PRJNA880768. We concatenated 20 single-copy alleles with dN/dS values <1 using autoMLST. Mapping of sequence reads to this 18,408 bp concatenated scaffold yielded average coverage of 104 – 282x across samples. The 11 isolates sequenced from menhaden were 100% identical across these 20 loci (18,408 bp). Strain CNEVA NB11008 had the closest identity to these isolates and differed by 2 bp across the concatenated sequence. These SNPs were present in the tRNA pseudouridine (55) synthase TruB and 1-deoxy-D-xylulose-5-phosphate synthase genes. Phylogenetic analysis identified nearest neighbor relationships in which the menhaden isolates and CNEVA NB11008 comprised a single clade (Figures 7 and 8). Average nucleotide identity across chromosome 1 yielded similar results, indicating that while most of the menhaden isolates were most similar to each other, they were most similar to CNEVA NB11008 compared to other publicly available sequences (Figure 9).

### 3.7. Experimental Infection of Atlantic Salmon with V. anguillarum

**Figure 10:**
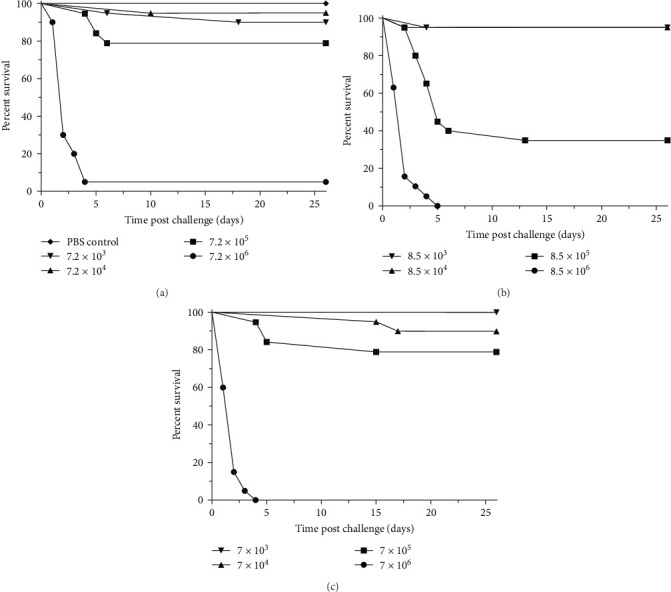
*Salmo salar* survival after exposure to *V. anguillarum*. Groups of 20 fish per dose tested were exposed by I.P. injection to the following strains: Va210524B (a), Va210001B (b), and Va201601B (c). Doses used are indicated in the graph inserts and are expressed as CFU injected per fish. A single group was mock exposed by injection with PBS and is shown in the top panel.

Because *V. anguillarum* is an important pathogen of farm-raised salmon, we performed laboratory challenge experiments to examine the risk that menhaden-associated strains pose to Atlantic salmon. With all three strains tested, injection exposure resulted in >95% mortality at the highest doses used (approximately 8 × 10^6^ CFU/fish) over the 28-day challenge period, with mortality starting at day 3 and ending at day 18 post-challenge (Figure 10). Lower doses also induced mortality, but to a lesser extent and in a dose-dependent manner. Moribund and dead fish exhibited gross clinical signs consistent with bacteremia induced by *V. anguillarum*, and the challenge bacteria were readily re-isolated from challenged fish that had died. These results demonstrate that the menhaden-associated strains have the potential to cause fatal infection in healthy Atlantic salmon when delivered by injection.

## 4. Discussion

We show that *V. anguillarum* was consistently isolated from fish exhibiting signs of spinning disease from three different mortality events between 2020 and 2021. Despite testing these fish on cell lines previously reported to be susceptible to a reported aquabirnavirus, including CHSE-214 cells and BF-2 cells [9], these failed to detect a viral etiology in our screening efforts. The temperatures ranging from 6.5–12°C around the start of epizootics in spring and fall are not typical for outbreaks with *V. anguillarum* in finfish. It is generally believed that disease by *V. anguillarum* occurs after water temperatures exceed 15°C [10]. Though other mortalities of wild fish have also documented vibriosis at low temperature, for example, mortality of wild big-scale sand-smelt *A. boyeri* in Greece occurred when water temperature was 11–12°C [13]. Furthermore, psychrotrophic strains of *V. anguillarum* have been described [26], and *V. anguillarum* was shown to possess a glycine betaine synthesis system that responds to cold stress and allows the bacterium to remain virulent in cold temperatures [27]. This may explain the virulence of *V. anguillarum* in menhaden during cool temperatures. It should be noted that the menhaden *V. anguillarum* grew most effectively *in vitro* at 25°C, with similar growth noted at 37°C, so optimal growth did not match temperature at the time of mortality. This is not unexpected since *V. anguillarum* adjusts the expression of virulence factors based on temperature; for example, one strain has been shown to have optimal growth at 25°C, while virulence was highest at 15°C [28]. In addition to bacterial virulence factors, another contributing factor may be related to host immune competence, which may be compromised at cool temperatures, as has been generally shown in various fish species [29].

The neurological (spinning) signs in the affected menhaden and the clear tropism for the brain are unique from previous descriptions of vibriosis caused by *V. anguillarum*, which typically causes hemorrhagic septicemia. Signs of disease have been reported to include skin hemorrhage, ulcers, skin discoloration, and erythema at the base of the fins, around the vent, and the mouth [10]. Other manifestations of *V. anguillarum* also include skin and muscle infections causing ulcers, fin rot, and anemia, as has been described for the winter flounder *Pseudopleuronectes americanus* [30]. Presently, we are not aware of other reports showing neurologic manifestation or brain infection of *V. anguillarum* in fish; thus, it is curious to see this as the most widespread finding associated with these menhaden mortalities. This finding may be explained by various factors, including host differences in pathogenesis, chronic infections which have been cleared systemically with persistence in the brain, and/or disease manifestation during cool temperatures. Other bacteria have been shown to cause persistent infection at high levels in the brain of fish, including *Weissella ceti* [31] and *Y. ruckeri* [32]. The possibility of systemic clearance and brain persistence was partly supported here. Fish in the early stage of mortality in March 2021 displayed signs of systemic disease with high levels of bacteria isolated from both the kidney and brain, whereas this population sampled nearly 1 month later had an absence of systemic disease and had high bacterial levels mainly limited to the brain.

Efforts were made to preserve histological material quickly after death, though logistics of locations caused preservation of tissues to occur from 2 to 15 hr postmortem. It is possible that pyknosis and necrotic changes in the kidney could have been influenced by autolysis, considering the high levels of bacteria and the kidney being sensitive to autolytic change. Previous studies in other fish species have shown that autolytic changes are minimal within 24 hr after death when fish are kept cool [33], though the gut, gill, kidney, pancreas, and liver are more prone to autolytic changes than other organs [34]. There was no detected autolytic change in the brain, and tissue morphology looked similar between all sampled material despite the timing of fixation following euthanasia (2–15 hr). Future experimental infections in menhaden will be helpful to better understand the pathogenesis and progression of *V. anguillarum* in this species.

We determined that *V. anguillarum* associated with menhaden kills belonged to serogroup O3. Serotyping is important for characterizing *V. anguillarum*, and the serogroup is indicative of fish-pathogenic strains. O-serotyping has identified 23O-serogroups, with O1–O3 being pathogenic in fish and the remaining being environmental isolates or ones causing only opportunistic infections in fish [35, 36]. Serogroups O1 and O2 are considered most pathogenic in certain fish species. Serogroup O1 was the predominant pathogenic serovar in other marine fish species, including European sea bass *Dicentrarchus labrax*, sea bream *Sparus aurata*, and mullet *Mugilidae*, whereas serogroup O2 was dominant in strains pathogenic to Atlantic cod *Gadus morhua* [37]. Though less frequently pathogenic in fish, serogroup O3 *V. anguillarum* is mainly known to be pathogenic to eels *A. anguilla* [37] and occasionally to rainbow trout *O. mykiss* [38]. Serotype O3 *V. anguillarum* appears to be limited in its distribution and is only occasionally isolated from diseased fish in Europe and Asia. However, in 2004, this serotype was identified as causing high levels of mortality in farmed Atlantic salmon, Pacific salmon, and rainbow trout in Chile [39]. These outbreaks represented the first isolation of *V. anguillarum* in Chilean salmonid aquaculture and suggested the recent emergence of serotype O3 in that region.

Molecular typing by WGS and MLST on menhaden isolates showed that all *V. anguillarum* isolates, representing a wide spatial and temporal scale in the mortality events, were the result of clonal expansion of a single strain. This suggests that a pathogenic strain is transmitting and circulating within the population, rather than opportunistic infections independently arising in individual fish, which would show more molecular diversity in the isolates. Notably, these isolates shared the closest identity to the virulent serotype O3, CNEVA NB 11,008 strain of *V. anguillarum* that was isolated from European sea bass in France in 1997. Differences were noted across the genome in terms of predicted CDS across both chromosomes 1 and 2, but this may simply be an artifact of the *ab initio* annotation methods of the partial genomes.

An unexpected finding was the sporadic isolation of *Y. ruckeri* along with *V. anguillarum* and the rare isolations of pure cultures of *Y. ruckeri* from the brain and kidney of dying menhaden. This has long been known as a bacterium specific to salmonids, though data have shown that other fish species, including marine fish, such as Atlantic cod [40], European sea bass, and turbot *Scophthalmus maximus* [41], may also be affected. Considering the sporadic detection compared to *V. anguillarum*, it is probable that *Y. ruckeri* had a more limited role in the menhaden mortality, though co-infections contributing to disease are likely. Despite its less frequent occurrence, the disease associated with *Y. ruckeri* should be considered in menhaden. Several fish had pure isolations of *Y. ruckeri*, and one fish yielded high bacterial loads and had moderate multifocal granulomas along with multifocal hepatic hemorrhages, a pathology that is similar to a report of yersinosis in Atlantic cod [40]. The detection of this agent in menhaden highlights a biosecurity concern for salmonids, particularly since these belonged to serotype O1, which are associated with high virulence in rainbow trout and Atlantic salmon internationally in areas where these species are farmed intensively [42–44]. The finding of *Y. ruckeri* in marine fish species is surprising, as this pathogen has been primarily associated with disease in the freshwater environment. *Y. ruckeri* does occur in sea-farmed salmon in Norway; however, it is unclear whether fish infected during the freshwater culture phase break with disease once in seawater or if true fish–fish transmission occurs in the seawater [45]. Some studies have indicated survival of *Y. ruckeri* in higher salinities, albeit at a shorter period when compared to freshwater [46], and in rainbow trout, enteric redmouth disease occurs, though it is less severe at increasing salinities [47].

Considering that menhaden are reservoirs of *V. anguillarum* and *Y. ruckeri*, their migration to the Gulf of Maine raises biosecurity concerns for the Atlantic salmon aquaculture industry. Furthermore, our experimental challenge results demonstrate a potential for the menhaden *V. anguillarum* strains to infect Atlantic salmon, though this was only tested via injection. Therefore, the migration of menhaden along the eastern Atlantic could provide a potential route for the transmission of these bacteria to Atlantic salmon aquaculture facilities in the northeastern United States and Canada. This is especially concerning given that the *V. anguillarum* vaccines currently used in these areas are designed to protect against serotypes O1 and O2 and are not expected to provide protection against serotype O3 strains. Further experimental work will be necessary to better evaluate the risks of menhaden *Y. ruckeri* strains to salmonids. With the promise of expanding marine offshore aquaculture in the United States to include other marine finfish species [5], making scientifically informed decisions regarding fish health management is vital for preventing disease outbreaks in the industry. Most beneficial will be practices that aid in the prevention of spillover of these bacterial agents from wild menhaden into farmed populations within a shared marine environment. In addition to natural migratory movements throughout their range, menhaden support a commercial bait industry ranging between 43,900 and 63,900 mt annual landings between 2012 and 2017 [4]. The findings of *V. anguillarum* and *Y. ruckeri* associated with menhaden mortalities would suggest that basic precautions should be taken within the bait trade to avoid the movement of these agents to sensitive areas, such as those that support aquaculture.

## Figures and Tables

**Table 1:** Cell culture surveillance of moribund *B. tyrannus* for virus detection from chronic mortality events.

Collection date	Collection location	# of fish	Cell lines/Temp	Cell culture results
June 10, 2015	Peconic River (7)	3	EPC; CHSE-214; BF-2/15°C	Negative (cytotoxicity)
May 24, 2016	Navesink River (2)	12	EPC; CHSE-214/15°C	Negative
May 3, 2017	Navesink River (2)	5	EPC; CHSE-214/15°C	Negative
June 28, 2018	Raritan River (near 3)	3	CHSE-214/20°C	Negative
May 1, 2020	Raritan River (near 3)	5	EPC; CHSE-214; BF-2/15°C	Negative (cytotoxicity)
May 12, 2020	Navesink River (2)	2	EPC; CHSE-214; BF-2/15°C	Negative (cytotoxicity)
Nov 30, 2020	Lower Hudson River (1)	9	EPC; CHSE-214; BF-2/15°C	Negative
Dec 1, 2020	Liberty St Park (1)	30	EPC; CHSE-214; BF-2/15°C	Negative
April 26, 2021	Navesink/Shrewsbury Rivers (2,5)	20	EPC; CHSE-214; BF-2/15°C	Negative

Collection location site numbers correspond to Figure 1.

**Table 2:** Summary of menhaden collection dates, locations, sample sizes, water quality, and fish biological data.

Date	Collection location	# Fish sampled	Water temp (°C)	DO (mg/L)	Mean TL ± SD (range) (cm)	Mean weight ± SD (range) (g)	Mean *k*-factor (range)
Dec 01, 2020	Liberty St Park (1)	30	11.5	9.0	29 ± 1.9 (24.5–33)	229 ± 32.6 (176–313)	0.92 (0.8–1.18)
Mar 30, 2021	Navesink River (2)	5	*⁣* ^*∗*^	*⁣* ^*∗*^	32.4 ± 1.2 (31–34)	292 ± 11.5 (287–313)	0.89 ± .08 (0.81–1.0)
Mar 30, 2021	Natco Lake (3)	7	*⁣* ^*∗*^	*⁣* ^*∗*^	33.4 ± 1.9 (31.3–36)	301 ± 22.7 (284–329)	0.82 ± 0.1 (0.72–1.0)
Apr 13, 2021	Great South Bay (4)	8	12.3 ± 0.5	7.6 ± 0.7	29 ± 1.5 (26.7–30.6)	*⁣* ^*∗*^	*⁣* ^*∗*^
Apr 22, 2021	Natco Lake (3)	6	10.8	8.0	30.8 ± 3.2 (24.5–32.5)	282 ± 54.5 (231–377)	0.80 ± 0.07 (0.71–0.87)
Apr 22, 2021	Navesink River (2)	6	11.0	8.0	32.5 ± 1.9 (30.3–35.5)	296 ± 40.1 (280–345)	0.86 ± 0.06 (0.85–0.93)
Apr 26, 2021	Shrewsbury River (5)	10	13.0	9.6	32.4 ± 1.9 (32–34.8)	308 ± 58.2 (252–391)	0.93 ± 0.09 (0.80–1.10)
Apr 26, 2021	Navesink River (2)	10	12.8	10.2	32.5 ± 1.2 (30.8–33.8)	294 ± 36.9 (235–339)	0.86 ± 0.1 (0.61–0.99)
May 10, 2021	Freeport Creek (6)	3	∼ 16.9	∼ 7.2	27.6 ± 2 (26.2–30)	*⁣* ^*∗*^	*⁣* ^*∗*^
May 12, 2021	Peconic River (7)	3	15.8	8	29.3 ± 0.2 (29.2–29.5)	*⁣* ^*∗*^	*⁣* ^*∗*^
May 12, 2021	Navesink River (2)	1	15.0	*⁣* ^*∗*^	36	421	0.90
May 12, 2021	Shrewsbury River (5)	5	15.0	*⁣* ^*∗*^	33.5 ± 1.4 (32–35.5)	289 ± 50.8 (220–347)	0.76 ± 0.08 (0.67–0.84)
May 17, 2021	Delaware Bay (8)	7	17.6	8.5	32.2 ± 1.8 (30.5–35.5)	251 ± 33.4 (212–313)	0.77 ± 0.05 (0.70–0.83)
Dec 28, 2021	Mill Pond, Mullica River (9)	5	6.5	9.8	27.9 ± 2.3 (25.5–30.5)	198.9 ± 37 (160.3–243.5)	0.91 ± 0.07 (0.85–0.98)

All collections were of moribund fish from mortality events in 2020 and 2021. Collection location numbers correspond to the map in Figure 1. Dissolved oxygen (DO), total length (TL), standard deviation (SD), condition factor (*k*-factor), and data not available (*⁣*^*∗*^).

**Table 3:** Summary of gross, bacteriological, and histopathologic findings from three different *B. tyrannus* mortality events off the coast of New Jersey.

	Finding	Fall 2020	Spring 2021 (E)	Spring 2021 (M)	Spring 2021 (L)	Fall 2021
Gross necropsy	Eye hemorrhage	6/30	0/12	10/32	4/12	1/5
	Skin/fin hemorrhage	18/30 L	4/12 L 3/12 S	16/32 L	3/12 L	0/5
	Anchor worm	18/30	9/12	7/32	9/12	5/5
	Brain congestion and hemorrhage	21/30	0/30	15/32	0/12	3/5
	Splenomegaly	0/30	7/12	0/32	0/12	0/5
	Multifocal liver hemorrhage	0/30	7/12	0/32	0/12	0/5

Bacterial isolation	*V. anguillarum*	2/2	10/12	26/32	8/12	5/5
	*Yersinia ruckeri*	0/2	0/12	2/12	3/12	0/5

Histology findings	Brain hemorrhage	23/30	2/12	10/12	2/6	0/3
	Encephalitis and necrosis	13/30	0/12	0/12	2/6	1/3
	Meningitis	13/30	0/12	10/12	2/6	1/3
	Bacterial rods in brain	2/30	0/12	2/12	2/6	0/3
	Renal hematopoietic necrosis	28/30	7/12	1/12	2/6	0/3
	Splenic congestion	9/30	7/12	3/12	0/6	2/3
	Splenic hematopoietic necrosis	11/30	0/12	0/12	1/6	0/3
	Hepatic perivasculitis	16/30	2/12	4/12	3/6 L	0/3
	Multifocal hepatic hemorrhage	0/30	2/12	0/12	0/12	0/3

In an ongoing mortality from the spring of 2021, three temporal samples were taken in the early (E), middle (M), and late (L) parts of the mortality event. For skin/fin hemorrhage, lesions were noted to be light (L) or severe (S).

**Table 4:** Number of sequence reads of the V3 and V4 regions of the 16S rDNA of the most abundant bacteria detected from brain (B) and kidney (K) tissue pools from moribund fish.

Bacterial species	B1	B2	B3	B4	K1	K2	K3	K4
*V. anguillarum*	37,470	39,144	40,198	33,437	76	99	13	249
*Wolbachia* sp.	440	218	221	931	2,127	4,598	3,220	4,495
*Bacteroides* sp.	270	126	53	555	1,352	4,212	2,128	2,110
*Faecalibacterium* sp.	48	66	49	242	1,366	349	2,565	986
*Pseudomonas* sp.	83	5	9	43	3,841	385	129	1,332

Percent identity within nucleotide sequence length was: *V. anguillarum* 99.8% (456 bp) multiple sequences, *W. pipientis* 99.3% (283 bp) Accession# CP037426, *B. vulgatus* 99.8% (454 bp) Accession# MT515977, *F. prausnitzii* 99.8% (434 bp) multiple sequences, and *Pseudomonas* sp. 99.8% (456 bp) multiple sequences.

## Data Availability

All sequence data are publicly available and associated with BioProject PRJNA880768 (https://www.ncbi.nlm.nih.gov/bioproject/880768). Additional data belong to the New Jersey Department of Environmental Protection, Fish and Wildlife Program (contact the Office of Fish and Wildlife Health and Forensics), and Stony Brook University (contact Bassem Allem at Bassem.allam@stonybrook.edu).

## Supplementary Materials

Supplementary Materials Figure S1: SDS-PAGE analysis of proteinase K-digested whole-cell lysates from 36 different *Vibrio anguillarum* isolates associated with *B. tyrannus* mortality between 2020 and 2021, visualized by Western blotting using antiserum raised against serotype 03. Lipopolysaccharide is normalized to culture optical density.
