# Supplementary material for: Seasonal Mortality of Wild Atlantic Menhaden (Brevoortia tyrannus) Is Caused by a Virulent Clone of Vibrio (Listonella) anguillarum; Implications for Biosecurity along the Atlantic Coastal United States
Source: Transbound Emerg Dis. 2024 Apr 12;2024:8816604. doi: 10.1155/2024/8816604 (PMC12017202; doi:10.1155/2024/8816604)
Supplement: Supplementary Materials — Figure S1: SDS-PAGE analysis of proteinase K-digested whole-cell lysates from 36 different Vibrio anguillarum isolates associated with B. tyrannus mortality between 2020 and 2021, visualized by Western blotting using antiserum raised against serotype 03. Lipopolysaccharide is normalized to culture optical density. [file 8816604.f1.pdf]

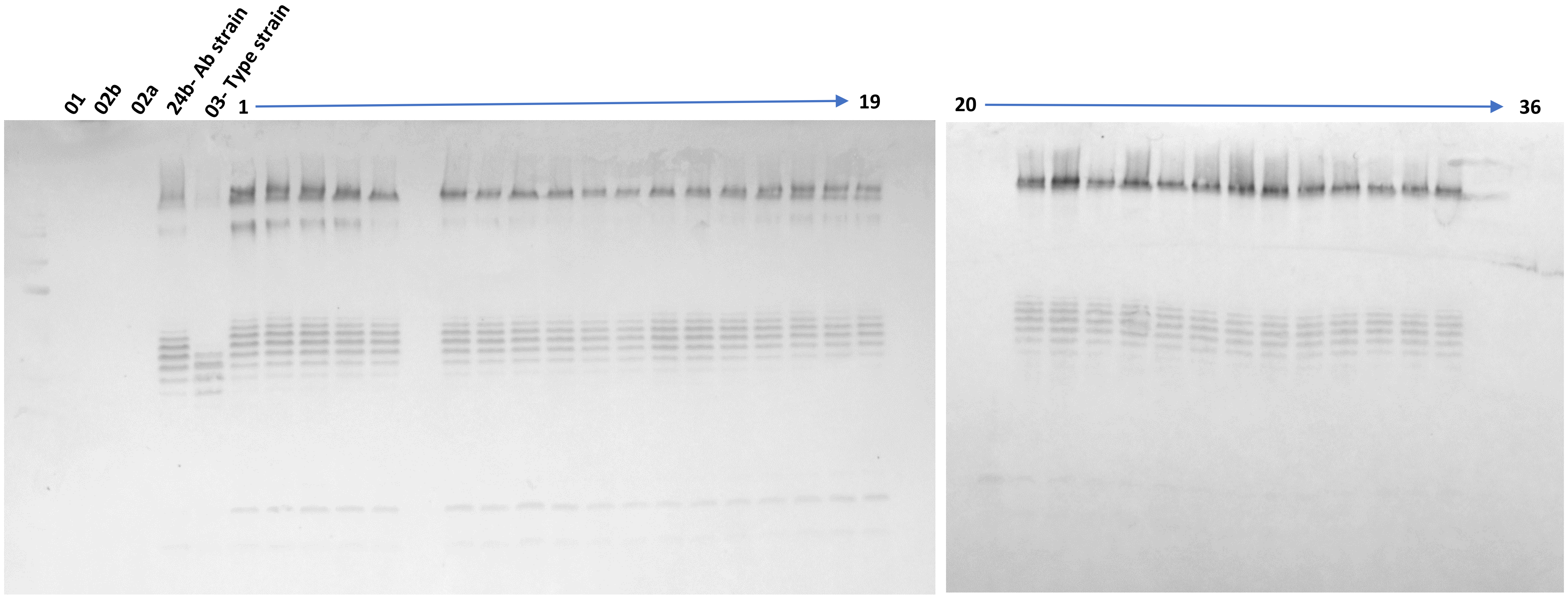

Lane #	ID	Collection Location
1	Va-21-2-2b	Navesink/Red Bank
2	Va-21-2-4b	Navesink/Red Bank
3	Va-21-2-7b	Natco Lake
4	Va-21-2-8b	Natco Lake
5	Va21-5-2B	Natco Lake
6	Va21-5-4B	Natco Lake
7	Va21-5-6B	Natco Lake
8	Va21-5-9B	Navesink/Red Bank
9	Va21-5-11B	Navesink/Red Bank
10	Va21-5-13B	Shrewsbury River
11	Va21-5-16B	Shrewsbury River
12	Va21-5-17B	Shrewsbury River
13	Va21-5-18B	Shrewsbury River
14	Va21-5-22B	Shrewsbury River
15	Va21-5-24B	Navesink River
16	Va21-5-28B	Navesink River
17	Va21-5-32B	Navesink River
18	Va-21-5-33B	Navesink River
19	Va-21-5-34Ka	Shrewsbury River
20	Va-21-5-35Ka	Shrewsbury River
21	7 21-018 2b-9	Freeport Creek
22	Va-21-5-42B	Delaware Bay
23	Va-21-5-43K	Delaware Bay
24	Va-21-5-45B	Delaware Bay
25	Va-20-16-1B	NJ
26	Va-20-16-6B	NJ
27	NYVa-1	Swan River
28	NYVa-8	Patch River
29	NYVa-10	Blue Point
30	NYVa-13	Blue Point
31	NYVa-20	Tuthils Creek
32	NyVa-22	Tuthils Creek
33	9 21-018 3b-2	Freeport Creek
34	11 21-019 2b-1	Peconic River
35	12 21-019 3b-1	Peconic River
36	MMSMS M21060803-K4	
